# Lack of benefit from low dose computed tomography in screening for lung cancer - comment on paper by Huang K-L et al.

**DOI:** 10.1186/s12890-020-01252-1

**Published:** 2020-08-26

**Authors:** Don Benjamin

**Affiliations:** Cancer Information & Support Society, 6/56 Chandos St, St Leonards, NSW 2065 Australia

**Keywords:** Lung cancer screening, Low dose computed tomography, Methodology

## Abstract

The article by Huang K-L et al. Effects of low-dose computed tomography (LDCT) screening on lung cancer contains a conclusion that is not consistent with the data presented. With reference to the National Lung Screening Trial (NLST) there are several flaws in the methodology overlooked. Also there is no significant reduction in deaths from all causes following the screening. Therefore any claim that the LDCT screening is superior to usual care is invalid.

## Main text

You recently published a paper by Huang K-L et al. entitled “Effects of low-dose computed tomography on lung cancer screening: a systematic review, meta-analysis, and trial sequential analysis” [1]. In that paper the authors state in their Conclusion that “LDCT screening has shown statistically significant mortality benefits in high-quality trials”. In the abstract they further state that “LDCT screening is superiority over usual care in lung cancer survival.”

Yet in the Section "Benefits and adverse outcomes" they state "On the contrary, LDCT screening demonstrated no statistically significant difference in all-cause mortality (RR 0.95, 95% CI 0.90–1.00).

The authors need to explain how a screening technique that produces no statistically significant difference in all-cause mortality between LDCT screening and usual care can be superior to usual care.

The authors also assess the risk for the NLST trial as being Good (Green +) on all criteria, including Methodology.

## Potential flaws in methodology

In fact the NLST trial had several methodological flaws related to the randomisation process overlooked by the authors of the paper:

1. The NLST trial compared LDCT screening of high risk smokers with Chest X-ray (CXR) screening and assumed that Chest X-ray screening produced the same outcome as usual care [2], as suggested in the Prostate, Lung, Colorectal and Ovarian (PLCO) Trial [3], despite earlier trials showing it resulted in an increase in all-cause mortality [4].
2. Anticipating the shortcoming in 1 above, the authors of the NLST trial ensured that the PLCO trial had, in addition to comparing average risk smokers, selected high risk smokers who were offered Chest X-ray screening for comparison with high risk smokers offered usual care - to validate the assumption referred to in 1. Yet this selection of high risk smokers was done after randomisation, so the comparison of deaths of high risk smokers after Chest X-ray screening with deaths of those receiving usual care was invalid. In addition the PLCO trial published only lung cancer deaths for the NLST-eligible high risk smokers, not deaths from all causes. This means the assumption in Point 1, that Chest X-ray screening of high risk smokers produced the same outcome as usual care in terms of all-cause mortality was invalid;

## Other irregularities

Reich and Kim observed that the distribution of deaths over time from the NLST-eligible groups selected from the PLCO trial showed irregularities, suggesting that there were some reporting errors in the PLCO trial. They also observed that there were no extra tumours found by the screening in the NLST-eligible groups selected from the PLCO trial [5], casting further doubt on this selection process, suggesting another flaw in the methodology. (The PLCO trial identified less than 5% more tumours by screening compared with about 20% more in previous chest X-ray trials.)

The above potential flaws and irregularities suggest that a ‘Red –‘ should be applied to the Randomization process, the Missing outcome data and the Overall risk rather than a ‘Green +’. On this basis a lower weighting should be applied to the NLST trial for the purposes of the meta-analysis.

The main shortcoming of the current meta-analysis, like that of many other randomised controlled trials (RCTs) is that the authors ignore the most important outcome, All-cause Mortality, and focus on the Deaths from Lung cancer. If there is no reduction in overall deaths following the screening, it is not valid to claim that LDCT screening is superior to usual care.

As pointed out by Black WC et al., All-cause Mortality in Randomized Trials of Cancer Screening, both trials of Chest X-Ray screening they reported on in 2002 showed an increase in all-cause mortality following Chest X-Ray screening that they attributed to the harm caused by post-screening treatments of higher risk smokers. They pointed out that as “disease-specific mortality may miss important harms (or benefits) of cancer screening because of misclassification in the cause of death, this end point should only be interpreted in conjunction with all-cause mortality. In particular, a reduction in disease-specific mortality should not be cited as strong evidence of efficacy when the all-cause mortality is the same or higher in the screened group” [4].

## Other issues

The NLST trial reported major complication rates following invasive procedures for the LDCT and CXR groups. The risk was higher among persons who underwent LDCT compared with Chest X-ray screening (4.1 vs 3.2 per 10,000 screened). The earlier CXR screening trials had shown an increase in deaths among those offered screening compared to those not offered screening (usual care). This is strong evidence in support of the suggestion that some of the reduction in deaths from lung cancer following LDCT screening could have been due to deaths from other causes resulting from the treatment that, as suggested by Black et al. above, should have been classified as deaths from lung cancer. There should therefore be strong reservations made about any claim that the LDCT screening was superior to usual care.

From the above, one possible explanation for the apparently positive result claimed in the NLST trial is that the Chest X-ray screening had in fact increased the number of deaths among those offered screening, as had been observed in previous trials [4]; the LDCT screening had reduced the number of deaths by a similar amount compared to Chest X-ray screening; the net result being that there was no significant reduction in overall deaths (as observed). Some of the reduction in lung cancer deaths could have been due to the methodological flaws outlined above.

Finally, the NLST trial is the only large trial to claim benefits for cancer screening, which would make lung cancer screening the only type of cancer screening to produce significant benefits. Randomised trials of breast, bowel, prostate and ovarian cancer screening have not produced significant reductions in all-cause mortality [6] and thyroid cancer screening has largely been discontinued due to much evidence suggesting no benefits but significant harm from overdiagnosis and overtreatment.

## Data Availability

Not applicable.

## Abbreviations

CXR: Chest X-ray
LDCT: Low dose computed tomography
NLST: National Lung Screening Trial
PLCO: Prostate, Lung, Colorectal and Ovarian (Trial)
RCT: Randomised Controlled Trial
RR: Relative Risk
CI: Confidence Interval
